# An economic analysis of chromosome testing in couples with children who have structural chromosome abnormalities

**DOI:** 10.1371/journal.pone.0199318

**Published:** 2018-06-19

**Authors:** Kittiphong Thiboonboon, Wantanee Kulpeng, Yot Teerawattananon

**Affiliations:** 1 Health Intervention and Technology Assessment Program, Department of Health, Ministry of Public Health, Muang, Nonthaburi, Thailand; 2 Centre for Health Economics Research and Evaluation, University of Technology Sydney, Haymarket, Sydney, Australia; Gettysburg College, UNITED STATES

## Abstract

**Background:**

Structural chromosome abnormalities can cause significant negative reproductive outcomes as they typically result in morbidity and mortality of newborns. The prevalence of structural chromosomal abnormalities in live births is at least 0.05%, of which many of them have parental origins. It is uncommon to predict structural chromosome abnormalities at birth in the first child but it is possible to prevent repeated abnormalities through screening and diagnostic programmes. This study will provide an economic analysis of the prenatal detection of these abnormalities.

**Methods:**

A cost-benefit analysis using a decision analytic model was employed to compare the status quo (doing nothing) with two interventional strategies. The first strategy (Strategy I) is preconceptional screening plus amniocentesis, and the second strategy (Strategy II) is amniocentesis alone. The monetary values in Thai baht (THB) were adjusted to international dollars (I$) using purchasing power parity (PPP) (I$1 = THB 17.60 for the year 2013). The robustness of the results was tested by applying a probabilistic sensitivity analysis.

**Results:**

Both diagnostic strategies can reduce approximately 10.7–11.1 births with abnormal chromosomes per 1,000 diagnosed couples. The benefit cost ratios were 1.62 for Strategy I and 1.24 for Strategy II. Net present values per 1,000 diagnoses in couples were I$464,000 for Strategy I and I$267,000 for Strategy II. The probabilistic sensitivity analysis suggested that the cost-benefit analysis was sufficiently robust, confirming that both strategies provided higher benefits than costs.

**Conclusions:**

Since the benefits of both diagnostic strategies exceeded their costs, both strategies are economical–with Strategy I being more economically attractive. Strategy I is superior to Strategy II because it decreases the risk of normal children potentially dying from the amniocentesis process.

## Introduction

Structural chromosome abnormalities (SCA) encompasses pathological alterations of the chromosome structure that results from the breakage or exchange of chromosome material [1]. Structural abnormalities are defined as unbalanced if there is a breakage or unequal exchange of the chromosome segment, and balanced if the complete chromosome set is still present even if it has been rearranged [2]. Unbalanced structural abnormalities typically have a dramatic impact on general health. Balanced structural abnormalities, however, exert no negative impact on health but they are of clinical significance due to their potential to adversely affect fertility and reproduce unbalanced structural abnormalities in the carrier’s children.

It has been estimated that–at minimum– 0.05% of newborns have unbalanced structural abnormalities [1]. Those born with abnormalities have a very poor chance of survival as up to 80–90% of babies die during their first year of life [3, 4]. For those who survive, structural abnormalities can result in intellectual deficits, and these individuals with retardation commonly have structural malformation that causes functional physical disability. Some children with SCA survive into adolescence and a few into adulthood; lifelong care is usually required from their families and the government.

Many structural chromosome abnormalities are interlinked from one of the parents, and some arise *de novo* (new to the individual). The certain likelihood that parents with structural chromosome abnormalities will reproduce a child with unbalanced abnormalities is unknown but it was approximated at between 9–13% depending on the forms of abnormalities [5]. It has been estimated that 37.50% of unbalanced structural chromosome abnormalities have parental origin [1].

It is difficult to distinguish a parent who carries a structural chromosome abnormality from those who do not. In many cases, the birth of the first child with unbalanced SCA from a balanced SCA couple is unforeseen. Some parents come to clinical attention for chromosome abnormalities after the birth of their first chromosomally unbalanced child. Identifying balanced SCA in couples with a previous unbalanced SCA child can make them aware of the possible risks associated with further pregnancies. When testing identifies SCA in the child, a chromosome analysis is recommended to be performed on both parents to investigate whether one of them has a unbalanced rearrangement [6]. For a pregnant woman whose baby is at high risk of SCA, prenatal diagnostic testing is recommended [7]. Whether or not chromosome analysis for carriers or prenatal diagnostic testing of the fetus is done, standard karyotyping is the gold standard technique [8].

This study aims to assess the costs and benefits of two policy strategies in order to prevent the recurrence of SCA births. This study was performed as part of the process for the development of the health benefits package under the Universal Health Coverage (UHC) in Thailand [9]. It is expected that the results of this study will empower the Thai government to adopt one of these policy strategies as well as inform other countries about the costs and benefits of each strategy.

## Methods

### Study design

This is a model-based economic evaluation study. A cost-benefit analysis was performed to evaluate two diagnostic strategies for SCA from a societal perspective; the analysis followed the standard guidelines of economic evaluation [10, 11], comparing two strategies with the status quo of not having a programme. Economic outcomes of interest were costs averted from having an abnormal child and couples’ willingness-to pay for the programme. A discount rate of 3% was applied for both future costs and outcomes [12]. All costs were calculated for the year 2013 and adjusted by the consumer price index [13]. All analyses were performed in Microsoft Excel 2007 (Microsoft Corp., Redmond, WA). Results were presented as benefit-cost ratios and net present values in international dollars (I$)–which was converted from Thai baht using the implied purchasing power parity of THB 17.60 per I$1 [14].

### Diagnosis strategies

In comparison with not having a programme, the analysis investigated two strategies to reduce the recurrence of births with SCA. Strategy I, which consists of preconceptional screening plus amniocentesis, is offered to all couples with a previous SCA birth who are planning for a subsequent pregnancy in order to investigate their carrier status. If couples with positive results still decide to go through with a pregnancy, an amniocentesis is given once the woman becomes pregnant. Strategy II, which consists of only an amniocentesis, is offered to all pregnant women with a previous SCA birth.

### Analytical model

**Fig 1:**
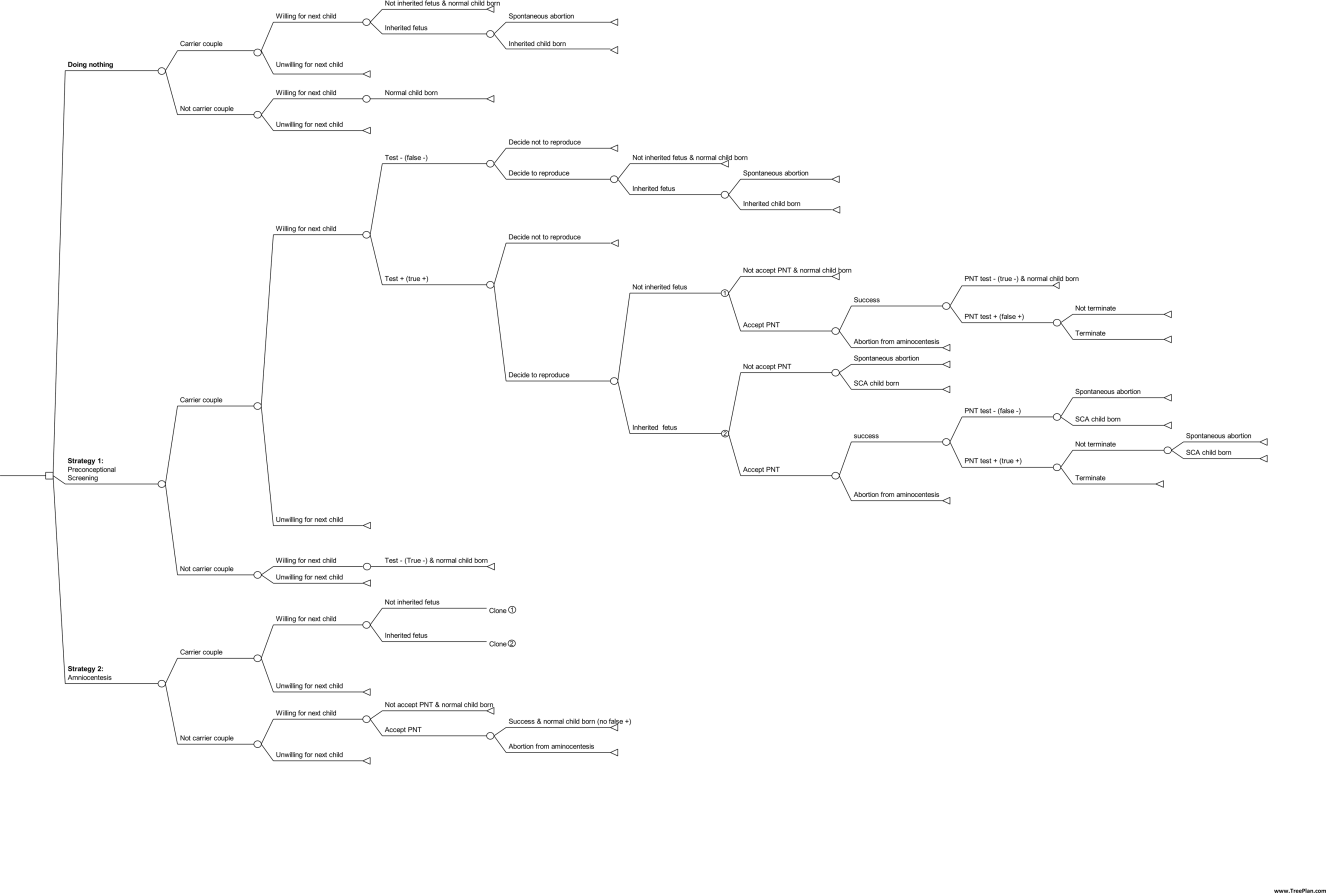
Decision tree of diagnostic strategies for the reduction of recurrent structural chromosome abnormalities in Thailand.

A decision tree was developed using Microsoft Excel, and the model structure is shown in Fig 1. This model estimates the costs and outcomes of Strategy I and Strategy II, and compares the results to the current practice. The model starts with the suspected SCA couple deciding to have another baby. A cohort of 1,000 couples with a previous SCA child was synthesised into the model to examine the relevant lifetime costs and benefits as well as other outcomes such as the number of abortions or normal child loss.

### Probabilities

About one-third of foetuses affected by SCA die during pregnancy. The remaining two-thirds continue to full-term but have high variations in terms of disease severity that require different degrees of care and treatment later in life. To allow for different treatment costs in this analysis, three groups were created according to disease severity as shown in Table 1.

**Table 1:** Categories of structural chromosome abnormalities (SCA) and important assumptions used in the analysis.

Category	Definition	Important assumptions
Category 1: Mild forms	Mental retardation, mild degree, no anomalies	- 10% of SCA - Average age is 58 years, same as a Down syndrome patient [38]. - Requires close care by parents until the age of 22 years. - Receives developmental and special education services.
Category 2: Moderate forms	Mental retardation with minor anomalies	- 45% of SCA - Average age is 18 years. - Requires close care by parents. - Does not receive developmental and special education services.
Category 3: Severe forms^1^	Mental retardation with major anomalies	- 45% of SCA - Average age is 5 years. - Requires close care by parents. - Does not receive developmental and special education services.

^1^Excluding spontaneous abortions and perinatal deaths

Due to the lack of studies on parents giving birth to a child diagnosed with SCA, an estimation of the probability associated with a woman's decision to become pregnant was based on the female carriers of Duchene muscular dystrophy in Thailand [15]. Based on that study, 71% decided to become pregnant with the knowledge that their previous child had a severe morbidity. In addition, 88% of couples who planned to conceive a child expressed a desire to have their chromosomes analysed after being informed that the investigation could detect chromosome abnormalities. Hence, the same rate of 88% was applied for parents who still expressed a desire to conceive (Table 2).

**Table 2:** Means and standard errors (SE) of input parameters.

Parameter	Distribution	Mean	SE	Data source
Probability				
*De novo* causes of SCA	Beta	.625	.084	[1]
Inheritance from carrier parent as unbalanced rearrangement	Beta	.085	.008	[5]
Spontaneous abortion	Beta	.381	.031	[18]
Willingness of parent with unknown carrier status to become pregnant (status quo)	Beta	.710	.160	[15]
Willingness of parent with unknown carrier status to become pregnant (diagnostic service provided)	Beta	.880	.110	[15]
of parent with unknown carrier status to become pregnant	Beta	.880	.110	[15]
Acceptance of amniocentesis by parent	Beta	.900	.040	[16]
Termination of pregnancy of unbalanced children	Beta	.890	.050	[39]
Amniocentesis procedure associated abortion	Beta	.005	.001	[19]
Sensitivity of traditional karyotype	Beta	.994	-	[40]
Specificity of traditional karyotype	Beta	1.000	-	[23]
Treatment access–age < 1 year	Beta	.300	-	Expert
Treatment access–age > 1 year	Beta	.150	-	Expert
Access to developmental and special education services		.663	.025	[25]
Costs–Chromosome analysis associated costs^1^ (I$)				
Blood chromosome analysis (per sample)	Gamma	118	9	[25]
Amniocentesis and chromosome analysis (per sample)	Gamma	236	19	[25]
Counseling cost (per time)	Gamma	41	4	[25]
Termination (per case)	Gamma	152	15	[25]
Direct non-medical costs for diagnosis (per time)	Gamma	88	12	[25]
Parental productivity cost (per time)	Gamma	51		[26]^2^
Costs–SCA patient treatment associated costs				
Lifetime direct medical cost of mild patients	Gamma	78,216		[25]^3^
Lifetime direct medical cost of moderate patients	Gamma	17,703		
Lifetime direct medical cost of severe patients	Gamma	21,322		
Lifetime direct non-medical costs of mild patients	Gamma	44,569		
Lifetime direct non-medical costs of moderate patients	Gamma	2,644		
Lifetime direct non-medical costs of severe patients	Gamma	1,050		
Lifetime parental productivity cost of mild patients	Gamma	56,112		[26]
Lifetime parental productivity cost of moderate patients	Gamma	128,321		[26]
Lifetime parental productivity cost of severe patients	Gamma	42,729		[26]
Normal child loss due to amniocentesis (per a case)	Gamma	204,787	5,451	[27]
Willingness to pay		2,895	774	[25]

^1^Costs in Thai baht (THB) were converted into the international dollar using the implied purchasing power parity (PPP) of THB 17.60 per international dollar (I$) in 2013.

^2^Calculated based on the assumption that a couple requires one day for the diagnosis process; the average wage per day was estimated from the GNI per capita.

^3^Re-estimated based on a previous economic evaluation.

SCA can be inherited from a parent or arise *de novo*; the latter accounts for 62.50% of abnormalities [1]. For parents with SCA, some of them conceive a child with SCA. From a previous cytogenetic study, the likelihood that a child inherits SCA from their parents ranged from 6% to 13% depending on the type of abnormality [5]. Thus, the midpoint of this range (8.5%) was used in the model as the base-case scenario.

The counseling process consisted of informing couples about the possible side effects of amniocentesis such as a slightly elevated risk of abortion. In a previous study [16], 90% of women at risk of conceiving a child with Down syndrome agreed to have further investigations performed to detect foetal abnormalities. This rate was applied to the study as the amniocentesis acceptance rate.

If an abnormality is detected in a foetus, the pregnant woman is given the choice to terminate the pregnancy or continue until birth. In Thailand, a relatively high number of pregnant women decide to terminate their pregnancy after being informed of abnormal test results. In one prospective study, 89% of women decided to terminate their pregnancy after learning that their foetus had a chromosome abnormality [17].

Two types of abortions were defined in this study: spontaneous and amniocentesis-related. The results of a study from the Netherlands were used in which 38% of carrier mothers with a subsequent pregnancy after two or more miscarriages had a spontaneous abortion [18]. The rate of procedure-related abortions used was 0.5%—which was obtained from a local study [19]; this was similar to the rate reported by the Centers for Disease Control and Prevention [20] and the American College of Obstetricians and Gynecologists (ACOG) [21].

The diagnostic sensitivity of karyotyping which was reported to be 99.4% according to the National Institute of Child Health and Human Development study in the US [22] was used in this study. The specificity of karyotyping was obtained from a UK study performed in 1,589 pregnant women in their second-trimester–which was reported to be 100% [23].

### Costs

Costs for performing conventional karyotyping for blood sampling and amniocentesis were I$118 and I$236, respectively [24]. The genetic counseling cost was estimated to be I$41 per session [25]. The total cost for transportation and meals was estimated to be I$88 per visit [25]. Productivity loss was derived from the average daily wage in Thailand and estimated to be I$51 per couple [26]. Mortality cost from the death of a normal child caused by amniocentesis was calculated by employing a human-capital method based on the official Thai working age of 15–60 years. The real Gross Domestic Product (GDP) growth estimated to be 3.50% per year, by the International Monetary Foundation (IMF) (ranging from 3.00%-3.90% between 2015 and 2023) [27] was used to account for labor productivity growth (a discount rate of 3.0% was applied). The cost of normal child loss due to amniocentesis was estimated at I$204,787 per child.

### Outcomes

The main outcome of the study was the cost averted by preventing the birth of child with SCA. The costs consisted of lifetime direct medical costs (drugs, and developmental and special education services), lifetime direct non-medical costs, and lifetime indirect costs (productivity loss due to morbidity and premature mortality). The outcome in terms of valuation of the diagnostic programme measured by the willingness-to-pay approach (WTP) was also considered [10]. A WTP of parents wanting to avoid the birth of a Down syndrome child in Thailand was estimated at I$2,895 and applied to the model [25].

Lifetime medical cost was estimated using treatment costs collected by the Central Office for Healthcare Information. From this database, the International Classification of Diseases, 10^th^ Edition (ICD-10) codes of Q92 and Q93 which were recorded as the primary diagnosis were then classified them into three groups according to medical expense. The data were further divided by age: less than one year and more than one year.

The cost of developmental and special education services was incorporated into medical costs. In Thailand, 66% of children with mental retardation have access to developmental and special education services [28]. In addition, it was assumed that only mildly retarded children participated in the programme. An analysis indicated that the lifetime medical cost of SCA patients ranged between I$17,703 and I$78,216.

In general, disabled persons require close care from their caregivers. In this study, it was assumed that those with mild disabilities needed close care until the age of 22. An analysis was performed by adopting data from a Thai study on Down syndrome [25] showing that the lifetime productivity loss of a parent would be I$56,112 per disabled child. For those with moderate and severe disabilities, it was assumed that they would need one caregiver on a full-time basis until the child's death. The productivity loss was calculated based on the same approach of estimating productivity loss in caring for a normal child. The estimated lifetime productivity loss of a caregiver with one disabled child was I$128,321 for the moderate type and I$42,729 for the severe type.

### Uncertainty analysis

To investigate the robustness of the benefit-cost ratio, a univariate sensitivity analysis was conducted for individual key parameters. The parameters were independently varied based on their distributional 95% confidence intervals. The most conservative and optimistic real GDP annual growth at 3.00% and 3.90%, respectively, were used to estimate a plausible range of costs of normal child loss due to amniocentesis, and the results were presented using a tornado diagram. The proportion of each disease severity (mild, moderate, and severe) was also modified from the base-case scenario and results were presented for comparison.

A probabilistic sensitivity analysis was also conducted to assess the uncertainty involving all model parameters. A Monte Carlo simulation was performed using Microsoft Excel 2007 (Microsoft Corp., Redmond, WA) to generate 1,000 samples drawn from the multivariate parameter distribution and present a range of possible costs, benefits, and benefit-cost ratios. Results were presented as a scatterplot of costs to benefits for each diagnostic strategy.

## Results

Considering a cohort of 1,000 couples with a previous child diagnosed with SCA, Strategy I and Strategy II were estimated to be able to prevent 11.1 and 10.7 births of children with chromosome abnormalities, respectively. Additionally, it was estimated that Strategies I and II would have resulted in 1 and 4 amniocentesis-related abortions, respectively.

Strategy I was estimated to yield a total cost of I$743,000 with a total benefit of I$1,207,000, resulting in a net benefit of I$464,000 and a benefit-cost ratio of 1.62. Strategy II exhibited costs of I$1,120,000 and benefits of I$1,387,000, yielding a net benefit of I$267,000 and a benefit-cost ratio of 1.24 (Table 3).

**Table 3:** Results of costs and benefits from Strategy I and Strategy II under base-case assumptions.

	Diagnostic strategy	
Result	Strategy I	Strategy II
Benefit (I$)	1,207,000	1,387,000
Cost (I$)	743,000	1,120,000
Benefit-cost ratio	1.62	1.24
Net benefit (I$)	464,000	267,000

**Fig 2:**
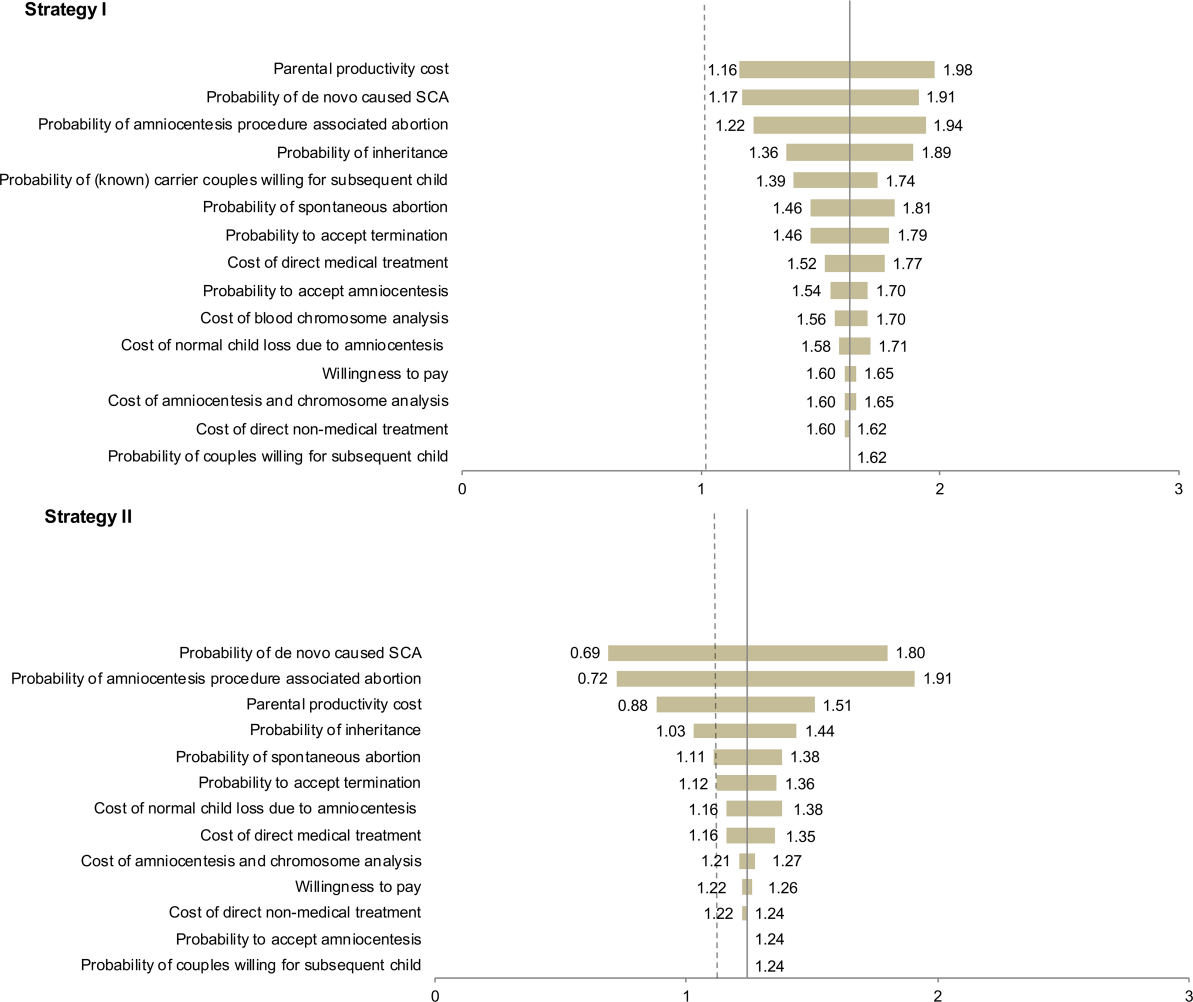
Tornado diagram showing the effect of varying each parameter on the benefit-cost ratio.

The results of the sensitivity analysis are illustrated in the tornado diagram shown in Fig 2. For Strategy I, the benefit-cost ratio was highly sensitive to parental productivity cost, proportion of *de novo* causes of SCA, and probability of amniocentesis-related abortions; however, the benefit-cost ratio was always greater than 1. For Strategy II, the benefit-cost ratio was mostly influenced by amniocentesis-related abortions (when the proportion of *de novo* is 69.61%, a benefit-cost ratio of Strategy II will be 1.00), proportion of *de novo* causes of SCA, and parental productivity cost.

Table 4 shows a comparison of benefit-cost ratios and percentage change from the base-case scenario for varying proportions of disease severities. It reveals that a greater proportion of children with mild abnormalities could lead to an increase in the benefit-cost ratio.

**Table 4:** Comparison of benefit-cost ratios and changes from the base-case scenario according to varying proportions of disease severity.

	Strategy I		Strategy II	
Proportions of disease severity (%) Mild:Moderate:Severe	BCR	% change	BCR	% change
10:45:45 (base-case)	1.62	-	1.24	-
30:35:35	1.73	6.79	1.32	6.45
50:25:25	1.83	12.96	1.40	12.90
70:15:15	1.93	19.14	1.48	19.35
90:5:5	2.04	25.93	1.56	25.81

BCR: Benefit-cost ratio.

**Fig 3:**
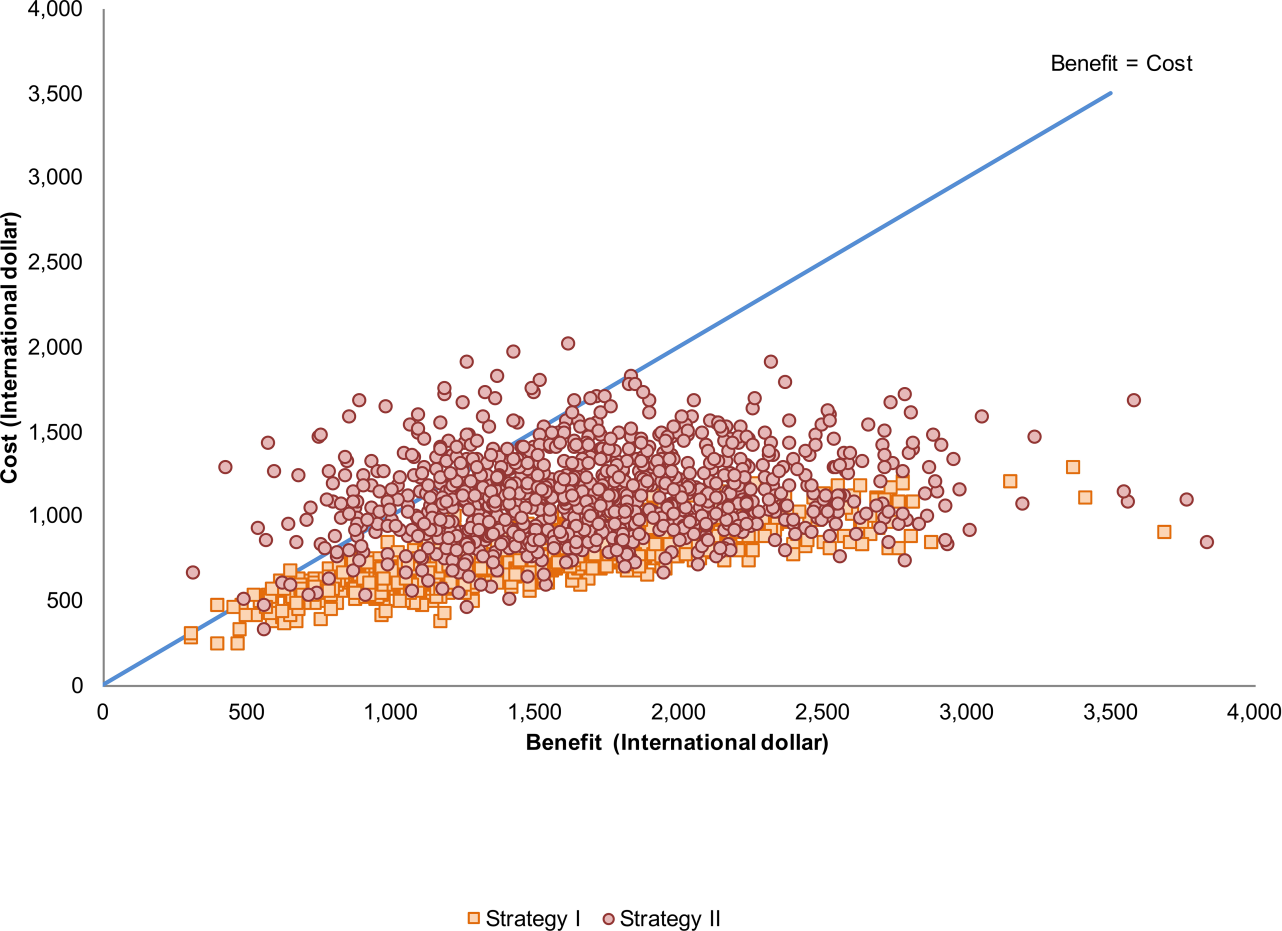
A scatterplot showing the results from probabilistic sensitivity analysis.

Fig 3 shows a scatterplot of benefits-to-cost over the distribution of Monte-Carlo simulations and demonstrates that there is very high probability that both diagnostic strategies are economical (benefit > cost). The figure also indicates that Strategy II has more probability than Strategy I to be uneconomical (benefit < cost).

## Discussion

Compared to the status quo, diagnostic services using either Strategy I or Strategy II resulted in positive economic outcomes. Strategy I was found to be the more attractive option compared to Strategy II. While a ceiling threshold for adopting health technologies evaluated using a cost-benefit analysis does not exist, this study reveals, at least, that the benefits of both strategies outweigh the costs. It is important to note that if decision-makers need to choose only one option, Strategy I might be the better alternative as it is less uncertain compared with Strategy II (Fig 3). However, it is still reasonable to provide prenatal testing for those who fail to have the carrier test since Strategy II was also economical. As a result, implementing either diagnostic service would be more economical than doing nothing at all.

Our findings are inconsistent with a prior study conducted in the Netherlands focusing on chromosome testing in women with a recurrent miscarriage to prevent handicapped children. That particular study found that performing an amniocentesis in women with a previous miscarriage was more economical than a parental chromosome analysis [29]. Reasons for the difference in results between the studies may be due to the different study objectives and methodologies used. For example, while the population of interest for this study was parents with an SCA child, the Netherlands study focused on women who had had a previous miscarriage. In addition, this study employed a cost-benefit analysis while the Netherlands study used a cost-effectiveness analysis.

Both strategies were sensitive to the proportion of *de novo* causes of SCA. Strategy II may become an uneconomical intervention if the proportion of *de novo* causes of SCA is more than 69.61%. However, it is believed that the base-case value for the proportion of *de novo* causes of SCA is 62.50%—based on a study which contained a large number of patients over a long period of time [1]. The benefit-cost ratios in both strategies were noticeably decreased as the risk of amniocentesis-related abortion increased. This resulted in a greater uncertain outcome in Strategy II as it could change the benefit-cost ratio to be less than 1.00. Parental productivity cost is another highly impact factor that is able to drop the benefit of Strategy II to below the cost.

Cost-utility analysis is now in high demand but it is not yet commonly used in areas of healthcare where benefits cannot be measured in terms of life-years gained or health-related quality of life [30]. This particularly includes antenatal care–where the abortion of the affected foetus and unborn disabled child are common measurements of effectiveness. In addition, while undertaking a cost-effectiveness analysis by expressing outcomes as cases averted is possible, such an analysis insufficiently reflects whether the programme is worthwhile, i.e. whether or not the benefits of the programme exceed its costs [10]. A cost-benefit analysis (CBA) provides an economic analysis of antenatal healthcare programmes by transforming health outcomes into monetary units [30]. However, the main criticism of the cost-benefit framework could be the principle that human lives and quality of life must be valued in monetary terms. Attempting to assign monetary values to health outcomes, particularly in the valuation of children, is controversial and has become a subject of debate between medical ethics and economics [31–34]. In particular, the concern about the human capital approach–which was used in this analysis–is that it tends to produce a lower value of life compared to other approaches used to place an economic value to a human life [35]. There is an argument that an unborn child should be recognised as having positive utility and value by any measure. By only accounting for the potential future costs of foetuses or unborn children without accounting for their potential to yield future benefits will create bias in the results. Hence, by omitting the valuation of such opportunity costs, this analysis is likely to provide a too-favourable result to the programme. These difficulties highlight why numerous decision-makers find this kind of policy question difficult or are not convinced by analyses which include these valuation problems [10].

Generalizability and transferability in which an economic evaluation is not completely interchangeable among countries is recognized in the decision-making procedures of health technology [36]. While several aspects of the study are generalizable and transferable to some countries, e.g. comparators, methodology, and parameters (e.g. abortion due to amniocentesis), others are not. One such issue is the amniocentesis procedure itself as it is not applicable to countries where termination is not permitted or even legal [37]. Without the ability to choose termination of the abnormal foetus, the strategy produces only costs without any potential benefits. In these settings, preconceptional screening would provide partial benefits in which couples will be provided with information about the risk of reproducing a child with abnormality, allowing them to make decisions on whether to avoid pregnancies with an abnormality. In countries where termination is permitted but not particularly chosen by couples, both strategies can still be options as it would enable some couples to avoid pregnancies with abnormalities. The sensitivity analysis suggests that even at the lowest possible value of the probability of termination, both strategies are still worthwhile–with preconceptional screening being more favorable compared an amniocentesis. Nevertheless, other countries might need to conduct their own assessment of the benefits against costs for the strategies to reflect the heterogeneities that differ from this study.

This study has a number of limitations which should be acknowledged. Firstly, due to limited epidemiological evidence regarding the variation in the severity of disease abnormalities, patients were categorised into three groups (mild, moderate, and severe). It is possible that the actual proportion of patients are different from the proportions used in the base-case scenario in this study. However, the results of the uncertainty analysis suggest that the results are not sensitive to changes in the proportions of these three groups.

Secondly, although this study used local data for estimating the probability associated with parental reproductive decisions, this parameter was derived from a study of women giving birth to children with Duchenne muscular dystrophy, not SCA. There may be a difference between Duchenne muscular dystrophy and SCA in terms of disease severity and probability of inheritance, resulting in different probabilities associated with a woman's decision to conceive. Results from the sensitivity analysis, however, indicated that this parameter did not seriously impact the results of the study.

Thirdly, some patients with mild SCA are likely to earn an income but these incomes were not included in the analysis; inclusion of income earned would reduce the estimated benefits of the analysis. However, a recent survey showed that the annual income among mentally retarded people is, on average, less than I$284, which is relatively low compared to Thailand's GNI per capita of I$9,058 [26].

## Conclusion

The results of this study suggest that reducing recurrent structural chromosome abnormality can be achieved by providing diagnostic health services such as parental chromosome analysis, and is economically attractive in Thailand. Investigating the carrier status of parents who have a history of structural chromosome abnormality in their child followed by an amniocentesis (Strategy I) is more economically attractive than conducting an amniocentesis alone (Strategy II). Preconceptional screening with an amniocentesis has advantages for parents as only carrier parents need to undergo this procedure. As a result, non-carrier parents can avoid this intrusive and dangerous process which carries a slight risk of death; it can also avoid possible errors of judgment or choice following the amniocentesis. Preconceptional screening might be useful in countries where termination is not well-accepted, prohibited or illegal.
